# The BJGP Open Top 10 Most Read Research Articles of 2025: an editorial

**DOI:** 10.3399/BJGPO.2026.0047

**Published:** 2026-03-19

**Authors:** Alexander Burrell, Hajira Dambha-Miller

**Affiliations:** 1 BJGP Open, Royal College of General Practitioners, London, UK

**Keywords:** Family medicine, Primary healthcare, General practitioners

*BJGP Open* has enjoyed another exceptional year, continuing to expand its reach and influence across the global primary care community. Submissions from 32 countries reflect both the vitality of contemporary general practice research and our commitment to providing an international platform for its dissemination. This year’s publications spanned a wide range of themes, from professional development in planetary health for African family physicians to the lived experience of dyslexia in UK GP training. Together, they demonstrate the breadth, intellectual curiosity, and social conscience that characterise modern primary care scholarship. In this editorial, we present our Top 10 Most Read Articles of 2025— articles that most captured the attention of our readership and, in doing so, shed light on the issues currently shaping general practice worldwide.

Having been absent from our 2024 Top 10, COVID-19 has returned to our 2025 list with The APC Collaborative Group’s article on the prevalence and severity of anxiety, stress, and depression among adults with long COVID in Barcelona.^1^ All three were widespread in the study population, although it was not possible to tease out cause and effect, with further work required to understand the pathogenesis of this complex condition. Another illness which can present a diagnostic challenge with diverse symptomology is Lyme disease: Delaney *et al* explored understanding, attitudes, and practice relating to this among primary care clinicians in the UK.^2^ They found important knowledge gaps around testing and treatment, with questions on prescription type and length frequently incorrectly answered.

Prescribing emerges as a prominent theme within this year’s list. Plehhova *et al* examined proton pump inhibitor (PPI) prescribing in England,^3^ highlighting substantial scope for optimisation. Despite widespread use of PPIs, many patients receiving continuous therapy had no recorded indication, and follow-up or medication review was frequently absent. These findings raise familiar concerns about therapeutic inertia and the challenges of deprescribing in routine practice. In contrast, medication with a clearer indication is antihypertensives: Dal Canto *et al* explored sex difference in the prescription of these medications in The Netherlands.^4^ Female patients tended to have lower daily dosage and more often received beta blockers and diuretics, receiving ACE inhibitors and calcium channel blockers less frequently. Their blood pressure was significantly better controlled. Continuing with chronic disease management, Ghosal *et al* conducted a systematic review and meta-analysis of the effectiveness of orlistat in type 2 diabetes.^5^ They found that overall mean weight loss with orlistat compared to controls was 2.40 kg (95% confidence interval CI = 2.08 to 2.72), with weight difference remaining statistically significant up to 12 months.

Good prescribing relies on accurate diagnosis: two of our top 10 studies look at testing in primary care. Carton *et al* conducted a feasibility study of the Montreal Cognitive Assessment in primary care in France to detect mild cognitive impairment, finding it was both feasible and easy to perform with a mean duration of eight minutes.^6^ Markwart *et al*’s multicentre observational study of point-of-care CRP testing in primary care in Germany found that this test was mainly used for respiratory tract infections, to distinguish between viral and bacterial aetiology and guide antibiotic prescribing decisions.^7^ This was felt to be a helpful test, improving confidence in treatment plans.

Providing good health care relies on individual staff and components of the health system engaging with developments and working well together. Jarvis *et al* studied engagement in liver disease management across Integrated Care Systems and health authorities across the UK.^8^ They found increasing engagement over time, but 36% of areas still had no liver pathways in place: more work is needed to reduce the postcode lottery. For patients who are admitted to hospital, good communication from secondary to primary care on discharge is key. Mikkelsen *et al* surveyed GPs on the use of marked discharge summaries in Denmark which must include specific follow-up actions required by the GP, colour-coded by urgency.^9^ These were felt to improve patient safety and post-discharge handover. In primary care, patients may see a range of staff, each of whom bring a different skillset. Eaton *et al*’s study of the impact of paramedics on primary care teams found that paramedics increased workforce capacity and health care availability, with successful integration relying on sufficient clinical experience and supervision from GPs.^10^

Our Top 10 Most Read Research Articles of 2025 reflect the breadth and vitality of contemporary primary care scholarship, spanning workforce, health systems, diagnosis, and prescribing. Collectively, they illustrate the range of our contributors and the practical concerns shaping everyday general practice across diverse settings. As we look to the year ahead, we warmly welcome further submissions addressing the pressing questions facing primary care. We are particularly keen to receive work exploring the evolving role of artificial intelligence and the interface between health and social care, themes that will be the focus of our forthcoming special issues.

## Data

N/A
